# Effect of Adenosine on the Morphological and Functional Integrity of Isolated Human Islets Exposed to Normoxic and Hypoxic Conditions

**DOI:** 10.3390/bioengineering13091053

**Published:** 2026-09-10

**Authors:** Heide Brandhorst, Daniel Brandhorst, Rebecca Spiers, Samuel Acreman, Lucian Christopherson, Anthony Cornu, Artjoms Portnojs, Keith Al-Hasani, Paul R. V. Johnson

**Affiliations:** 1 Islet Transplant Research Group, Nuffield Department of Surgical Sciences, University of Oxford, Oxford OX3 9DU, UK; heide.brandhorst@nds.ox.ac.uk (H.B.); rebecca.spiers@nds.ox.ac.uk (R.S.); samuel.acreman@ocdem.ox.ac.uk (S.A.); lucian.crawford@nds.ox.ac.uk (L.C.); anthony.cornu@nds.ox.ac.uk (A.C.); artjoms.portnojs@nds.ox.ac.uk (A.P.); keith.al-hasani@nds.ox.ac.uk (K.A.-H.); paul.johnson@nds.ox.ac.uk (P.R.V.J.); 2 Oxford Consortium for Islet Transplantation, Oxford Centre for Diabetes, Endocrinology, and Metabolism (OCDEM), Churchill Hospital, University of Oxford, Oxford OX3 7LE, UK; 3 Oxford Centre for Diabetes, Endocrinology and Metabolism, Radcliffe Department of Medicine, Oxford OX3 7LE, UK; 4 Epigenetics in Human Health and Disease Program, Baker Heart and Diabetes Institute, Parkville, Melbourne, VIC 3004, Australia; 5 Department of Cardiometabolic Health, The University of Melbourne, Parkville, Melbourne, VIC 3010, Australia

**Keywords:** adenosine, adenosine receptors, ADORA1, 2a, 2b, 3, human islets, hypoxia, islet apoptosis, islet mortality

## Abstract

Adenosine is a ubiquitous stress marker and is characterised by numerous vital functions in all organs. Although adenosine has been investigated for decades, the information about its metabolism in islet cells has mainly been obtained in rodents. The present study was undertaken to assess the effects of adenosine on isolated human islets when exposed to hypoxia. Isolated human islets were cultured in 1 or 5 mmol/L adenosine for 4–5 days at 1.5% oxygen. The postculture outcome was normalised to normoxia and 0 mmol/L adenosine. Whereas the mRNA expression of ADORA1, 2a, 2b and 3 in normoxia was unaffected by adenosine, hypoxia significantly increased the mRNA expression of these genes, which was further enhanced when islets were treated with adenosine. Hypoxia was the variable exerting the strongest impact, reducing islets’ morphological and functional integrity. These parameters were improved by adding 1 mmol/L adenosine but deteriorated when using 5 mmol/L. The harmful effects of adenosine on islet apoptosis and total mortality were dose-dependent and present in all atmospheres. Islet characterisation clearly demonstrated that hypoxia is the main stressor that decreases islets’ morphological and functional integrity. Further detailed studies are required to lock or unlock the different ADORA receptors and to identify their specific effects on islets’ morphological and functional integrity.

## 1. Introduction

Adenosine (ADO) is a signalling molecule that is involved in a broad range of vital functions within normal mammalian physiology and metabolism [1]. Its ubiquitous involvement in cell signalling, as well as in the generation and transfer of energy, makes ADO a key compound affecting all organs including renal, cardiovascular and neuronal tissue [1,2,3,4]. Beyond its essential role in islet physiology, ADO seems to contribute to glucose homeostasis by promoting the regeneration and proliferation of beta-cells [5]. It is remarkable that ADO was identified as the most potent enhancer of beta-cell regeneration in zebrafish amongst 7000 different compounds [6]. Another scientific opportunity has been found by introducing a zebrafish model with GATA6 mutations for human liver diseases [7].

ADO has been considered an essential key stress signal for human physiology [8,9,10] particularly relevant during episodes of ischaemia or hypoxia. A lack of oxygen is one of the most clinically relevant factors for tissue integrity and is a major cause of the dysfunction and failure of native and transplanted organs, with isolated pancreatic islets of Langerhans, used for transplantation for type 1 diabetes mellitus, being particularly sensitive to hypoxia [11,12,13,14,15]. In addition to this physiological burden, islets are exposed to enormous biophysical and biochemical stress during the isolation procedure, which substantially affects the functional and morphological integrity of isolated islets [15,16,17,18,19]. While ADO has been under investigation for nearly sixty years [1,20], our understanding about ADO metabolism in hypoxic isolated human islets is still very incomplete.

Previous studies indicated that cold storage in University of Wisconsin (UW) solution optimises the maintenance and preservation of human islet tissue until islet transplantation (TX) is performed. The addition of 1 mmol/L ADO increased islet yield even further but simultaneously decreased islet viability when used at a concentration of 5 mmol/L [21]. We hypothesised that this ambivalence might be explained by the simultaneous activation of different ADO receptors, namely ADORA1, 2a, 2b and 3. The mRNA expression of these ADO receptors had been investigated in different organs and tissues retrieved from various species, but the number of samples specifically collected from pancreas and islet cells remains small [22,23,24,25]. The knowledge and information about the character and expression of the ADO receptors ADORA1 to ADORA3 have been nearly exclusively collected in rodent islets.

As shown in Table 1, most of the information that had been gathered was related to ADORA1. The majority of data had been collected from mice and suggests that ADORA1 has an inhibitory effect on insulin secretion [26,27,28,29]. This observation could be confirmed in rat and human islets [30,31,32]. Samples collected for the characterisation of ADORA2a indicate a stimulatory effect on murine islet insulin release, which seems to depend on the concentration and the chemical character of the agonist used [28]. In addition, ADORA2a appears to have an anti-inflammatory potency which protects islet graft function in syngeneic mice [33]. Whilst only one single study had been performed for the assessment of ADORA2b, two experiments had been undertaken to investigate the influence of ADORA3. These findings imply that ADORA2b improves glucose homeostasis in a type 2 diabetes mellitus model [34] and that the activation of ADORA3 seems to reduce islet viability [28]. It is evident that the outcome of ADORA3 analysis is quite heterogeneous and depends on the concentration of the stimulatory agent used.

The aim of this study, therefore, was to try to narrow this substantial gap in knowledge and to provide a scientific and clinically relevant contribution to the ADO-specific knowledge in human islet tissue.

## 2. Materials and Methods

### 2.1. Human Islet Isolation

This study was approved by our institutional review board South Central—Oxford Research Ethics Committee (NHS Health Research Authority)—Oxfordshire Rec B (reference number 09/H0605/2, IRAS 215057; extension approval date: 27 January 2026). All donor pancreata were voluntarily donated after obtaining written informed consent from donor relatives according to the Declaration of Istanbul.

Pancreases were retrieved from six male human multi-organ Donors after Brain Death (DBDs) with a mean age of 49.2 ± 3.2 years (±standard error) and a mean body mass index of 25.8 ± 1.0 kg/m^2^. All pancreases were preserved with University of Wisconsin solution (Bridge to Life, London, UK) and had a mean Cold Ischaemia Time (CIT) of 6.1 ± 0.6 h. Human islets were isolated and purified using standard isolation techniques as previously described in detail [35].

### 2.2. Human Islet Culture

After isolation and purification, an average of 600 islet equivalents (IEQ) per well were placed in 12-well plates (Greiner Bio-One, Stonehouse, UK) and suspended in 1000, 950 or 750 µL of the culture medium CMRL 1066, respectively, corresponding to an average seeding density of 125 IEQ per cm^2^. The culture medium was supplemented with 20 mmol/L HEPES, 2 mmol/L L-glutamine, 200 units/mL penicillin, 200 µg/mL streptomycin (all reagents from Life Technologies, Paisley, UK) and 5% fetal calf serum (PAA Laboratories, Pasching, Austria). Afterwards, 0, 50 or 250 µL of a DMSO-dissolved (0.4%) 20 mM adenosine stock solution was added to obtain a final volume of 1000 µL per well and a final concentration of 0, 1 or 5 mM adenosine, respectively. Isolated islets were cultured at 37 °C for four to five days in normoxic conditions (21% oxygen [O_2_], 5% carbon dioxide, equivalent to a partial oxygen pressure (pO_2_) of approximately 160 mm Hg) or in a hypoxic atmosphere (1.5% oxygen, 5% carbon dioxide, equivalent to a pO_2_ of approximately 10 mm Hg).

### 2.3. Islet Characterisation

Before and after culture, samples of defined volume were collected from each treatment group and stained with dithizone (Sigma-Aldrich, Dorset, UK) to visually determine the number of islets defined as dithizone-positive size-independent insulin-containing cell clusters. Counted islets were categorised according to size and mathematically converted into islet equivalents (IEQ) considering the individual volume of counted islets as previously described in detail [36]. This conversion was normalised to a “standard” islet of 150 µm in diameter, which is defined as one IEQ. Islet morphological integrity was determined by calculating the Islet Fragmentation Index, dividing the number of size-independent actual islets (IN) by IEQ (Islet fragmentation Index = IN ÷ IEQ) [37]. Islet viability was assessed utilising 0.67 µmol/L of fluorescein diacetate (FDA, Sigma-Aldrich) and 4.0 µmol/L of propidium iodide (PI, Sigma-Aldrich) for the staining of viable and dead cells, respectively [38]. The fluorescence intensity (FI) of FDA-PI was quantified utilising a fluorometric plate reader as previously described [39]. The overall survival in IEQ was calculated considering the recovery of viable cells only, stained exclusively by FDA and not being penetrated by PI. Early apoptosis was demonstrated by the exclusive staining of phosphatidylserine using Annexin-V FITC (Becton-Dickinson Biosciences, Oxford, UK). In contrast, islet late apoptosis was determined by simultaneous staining with Annexin-V and PI used at concentrations of 450 ng/mL and 4.0 µmol/L, respectively [40,41]. Islet mortality was defined as the sum of islet early apoptosis plus islet necrosis (PI).

The in vitro function of 20 islets of similar size (150–200 µm) was assessed in duplicate during static glucose incubation. These islets were seeded on 8 µm pore size filter inserts, transferred into 24-well plates and sequentially incubated for 45 min in 1 mL of Krebs–Ringer buffer supplemented with 2.0 mmol/L glucose, followed by 45 min at 20 mmol/L glucose. After stimulation, islets were recovered and sonicated in 1 mL of distilled water. An aliquot of the disintegrated islet cell suspension was mixed with acid ethanol at a ratio of 1:4, followed by overnight insulin extraction at 4 °C [42]. Before performance of the human insulin-specific enzyme immunoassay (Mercodia, Uppsala, Sweden), samples were diluted and neutralised by using Krebs–Ringer buffer. The secretory capacity of stimulated islets was determined as the glucose stimulation index (GSI), which was calculated by dividing the insulin release at 20 mmol/L glucose by the basal condition at 2 mmol/L. Insulin release and intracellular insulin content were related to µU per IEQ.

### 2.4. Quantitative Real-Time Polymerase Chain Reaction

The gene expression of cultured islets (*n* = 6) was measured using TaqMan-based quantitative real-time polymerase chain reaction (qRT-PCR). Briefly, total RNA was extracted from 100 cultured handpicked islets of similar size (150–200 µm) using the RNeasy Micro kit (Qiagen, Manchester, UK) before being run in triplicate for 35 cycles on a QuantStudio 7 (Applied Biosystems, Warrington, UK) using the CellsDirect One-Step qRT-PCR kit (Invitrogen, Paisley, Scotland, UK). Duplex reactions were performed using TaqMan assays specific for the ADO receptor target genes 1 (ADORA1, Hs00181231_m1), 2a (ADORA2a, Hs00169123_m1), 2b (ADORA2b, Hs00386497_m1) and 3 (ADORA3, Hs00181232_m1) and normalised to 18S ribosomal RNA (rRNA) (18S rRNA, Rn01452893_g1). All primers were provided by Applied Biosystems (Warrington, UK). Quantitative values were obtained using the threshold cycle number and the x-fold change in expression using the ΔΔC_T_ method [43]. qRT-PCR data were normalised to ADO-untreated islets cultured in normoxia.

### 2.5. Statistical Analysis

Statistical analysis and graphical presentations were performed using Prism 11.0.0 (GraphPad, La Jolla, USA). Analysis of data was carried out using the nonparametric Friedman test followed by Dunn’s test for multiple comparisons. Correlation analysis was performed, calculating the nonparametric Spearman’s correlation coefficient (*r*). Where appropriate, data were normalised to sham-treatment. Differences were considered significant at *p* less than 0.05. *p*-values larger than 0.05 were termed nonsignificant (NS). The results are generally expressed as mean ± standard error (SEM).

## 3. Results

### 3.1. ADORA mRNA Gene Expression

**Figure 1:**
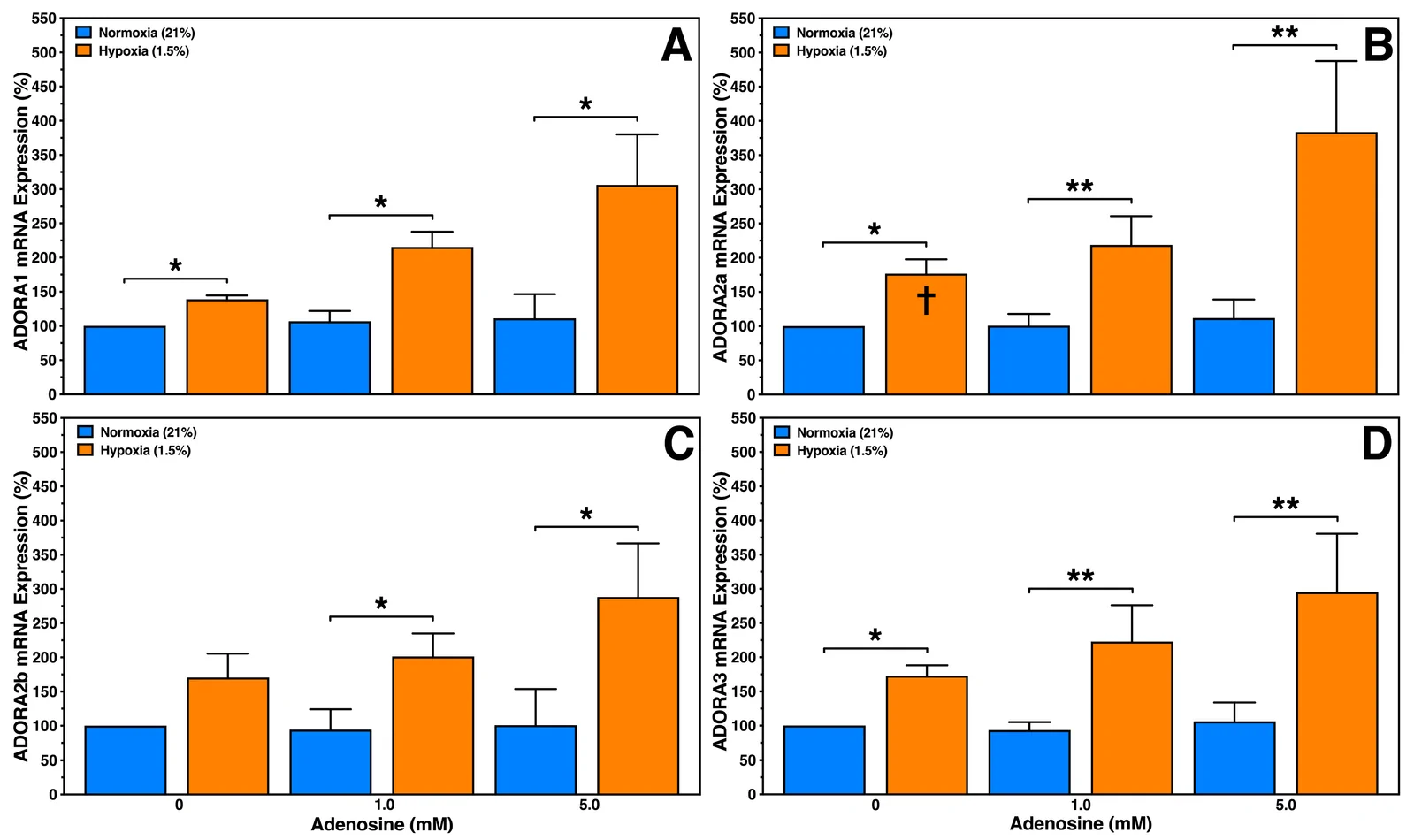
Effect of adenosine treatment on mRNA expression of the ADO receptors (**A**) ADORA1, (**B**) ADORA2a, (**C**) ADORA2b and (**D**) ADORA3 after 4–5 days of islet culture in normoxic (21% O_2_) or hypoxic (1.5% O_2_) atmosphere at 37 °C (*n* = 6). Outcome of qRT-PCR was normalised to control conditions at 21% O_2_ and 0 mmol/L ADO, and y-axes were harmonised to facilitate comparison of different genes. † *p* < 0.05 inside the bar indicates significance for ADORA2a expression in hypoxic islets treated with 5 mmol/L ADO. Lines/arrows indicate * *p* < 0.05 and ** *p* < 0.01, for comparison of normoxic vs. hypoxic islets.

As shown in Figure 1A–D, the mRNA expression of ADORA1–3 followed a nearly identical pattern. Equally, the extent of receptor expression was similar when comparing ADORA1 to 3. It became apparent that the highest mRNA expression was obtained when islets were exposed to hypoxia. Although not statistically significant, a clear trend towards increased expression of all four ADORA receptors was found when hypoxic islets had been treated with 5 mmol/L adenosine. In contrast, under normoxic conditions, an effect of ADO on ADORA mRNA expression was virtually absent.

### 3.2. Islet Morphological Integrity

**Figure 2:**
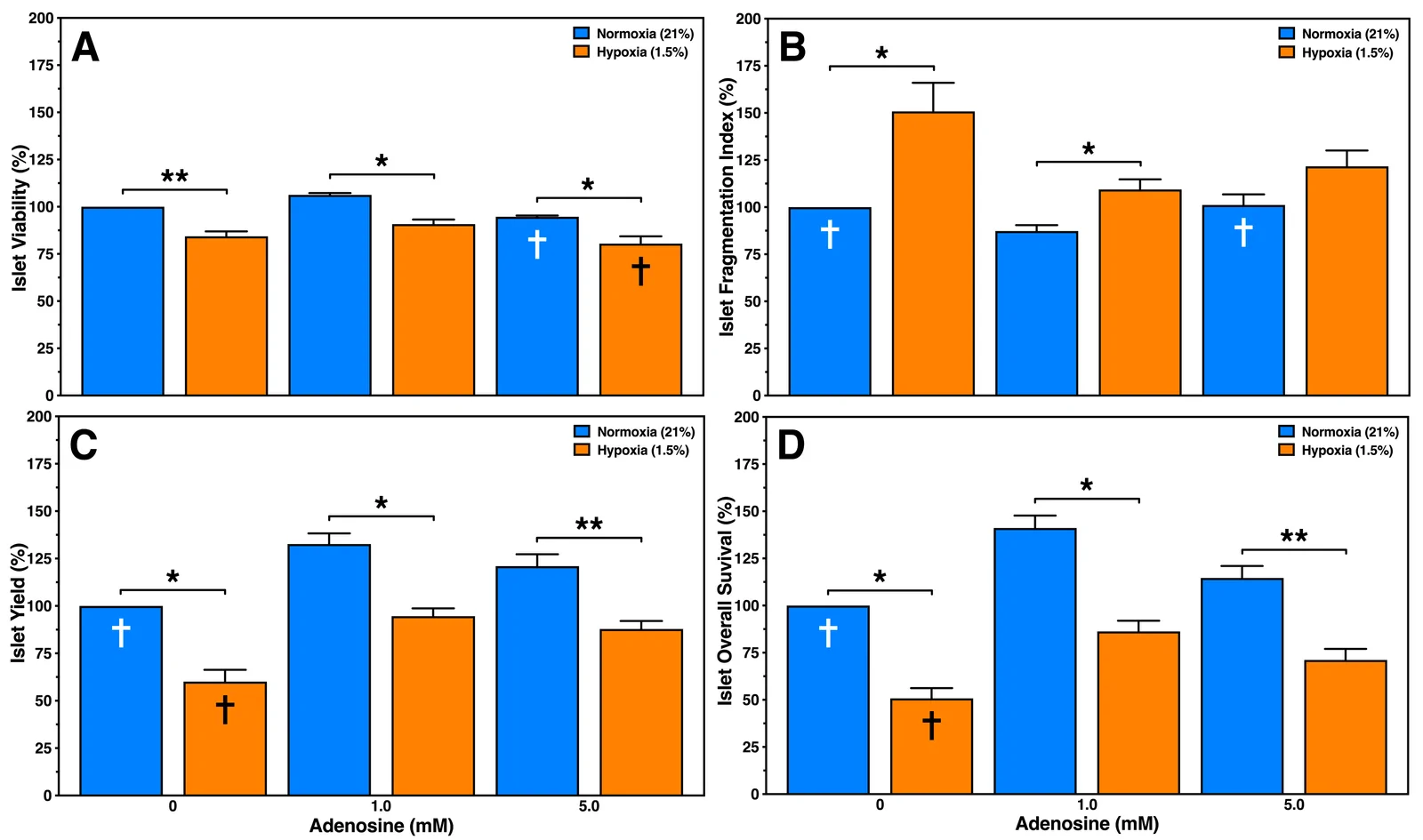
Effect of ADO treatment on morphological integrity of islets cultured for 4–5 days in normoxic (21% O_2_) or hypoxic (1.5% O_2_) atmosphere at 37 °C (*n* = 6). Islets were characterised in terms of (**A**) viability (%), (**B**) fragmentation (%), (**C**) postculture yield (%) and (**D**) overall survival (%). Postculture outcome was normalised to control conditions at 21% O_2_ and 0 mmol/L ADO, and y-axes were harmonised to facilitate comparison of different parameters. Symbols inside bars indicate † *p* < 0.05 for comparison of normoxic or hypoxic islets vs. corresponding islets treated with 1 mmol/L ADO. Lines/arrows indicate * *p* < 0.05 and ** *p* < 0.01 for comparison of normoxic vs. hypoxic islets.

Islet characterisation clearly revealed that hypoxia was the main determinant for islet loss in our experimental setting. This loss could be slightly reduced by adding 1 mmol/L of adenosine, which created an ameliorating effect with respect to viability (Figure 2A, † *p* < 0.05 5 mmol/L ADO) and islet morphological integrity (Figure 2B, † *p* < 0.05 vs. 0 and 5 mmol/L ADO). This effect was present under both normoxic and hypoxic conditions and resulted in significantly increased islet yield after culture (Figure 2C, † *p* < 0.05 vs. 0 mmol/L ADO). As a consequence, islet overall survival was significantly enhanced by approximately 40% and 60% after normoxic and hypoxic culture in 1 mmol/L ADO (Figure 2D, † *p* < 0.05 vs. 0 mmol/L ADO), respectively. When the ADO concentration was increased to 5 mmol/L, the protective effect of ADO was diminished.

### 3.3. Islet Mortality

**Figure 3:**
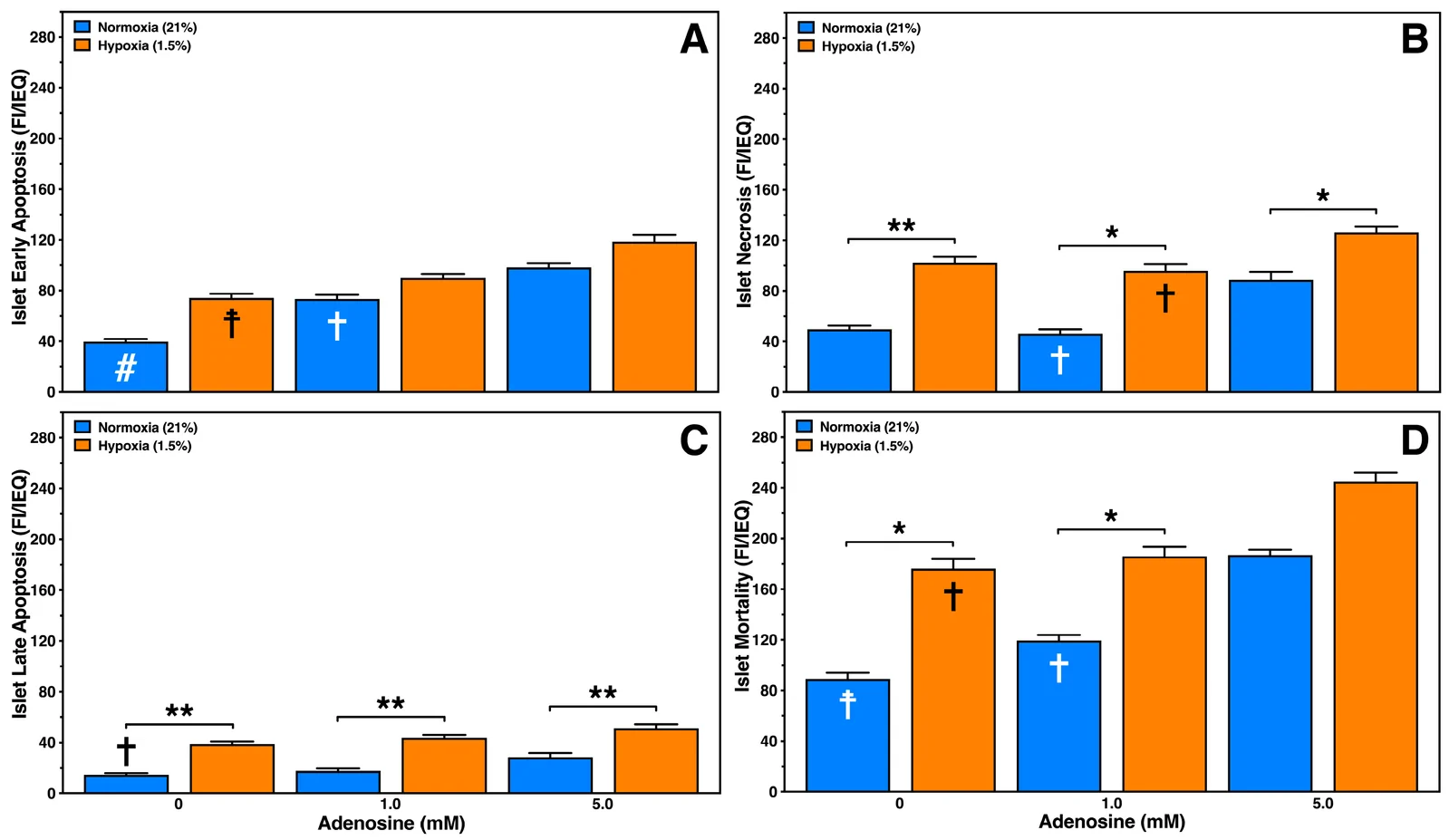
Effect of ADO treatment on different modes of islet cell death categorised as (**A**) early apoptosis, (**B**) necrosis, (**C**) late apoptosis and (**D**) mortality as observed in islets cultured for 4–5 days in normoxic (21% O_2_) or hypoxic (1.5% O_2_) atmosphere at 37 °C (*n* = 6). Postculture outcome was normalised to FI per IEQ, and y-axes were harmonised to facilitate comparison of different parameters. Symbols inside bars indicate † *p* < 0.05, ☨ *p* < 0.01, and # *p* < 0.001 for comparison of normoxic or hypoxic vs. corresponding islets treated with 5 mmol/L ADO. Lines/arrows indicate * *p* < 0.05 and ** *p* < 0.01 for comparison of normoxic vs. hypoxic islets.

To estimate the impact of ADO on islet morphological integrity, we analysed different modes of islet cell death. Compared with basal control conditions at 0 mmol/L ADO, islet early apoptosis increased dose-dependently in normoxia as well as in hypoxia when ADO was added (Figure 3A, † *p* < 0.05, ☨ *p* < 0.01, # *p* < 0.001 vs. 5 mmol/L ADO). A similar pattern of cell death was observed for islet necrosis despite the significantly stronger effect of hypoxia on this mode of cell death (Figure 3B, † *p* < 0.05 vs. 5 mmol/L ADO). The simultaneous cellular staining of cultured islets with Annexin-V and PI revealed that the proportion of late apoptosis was substantially lower compared with the early stage of apoptotic cell death. The highest level was reached at a concentration of 5 mmol/L ADO (Figure 3C, † *p* < 0.05 vs. 0 mmol/L ADO). In conformity with the other modes of cell death, it was demonstrated that 5 mmol/L ADO increases islet mortality (Figure 3D, † *p* < 0.05 vs. 0 and 1 mmol/L, ☨ *p* < 0.01 vs. 0 mmol/L ADO) Again, Figure 3D clearly shows that the lack of oxygen is an important stimulus for islet mortality.

### 3.4. Islet Secretory Capacity

**Figure 4:**
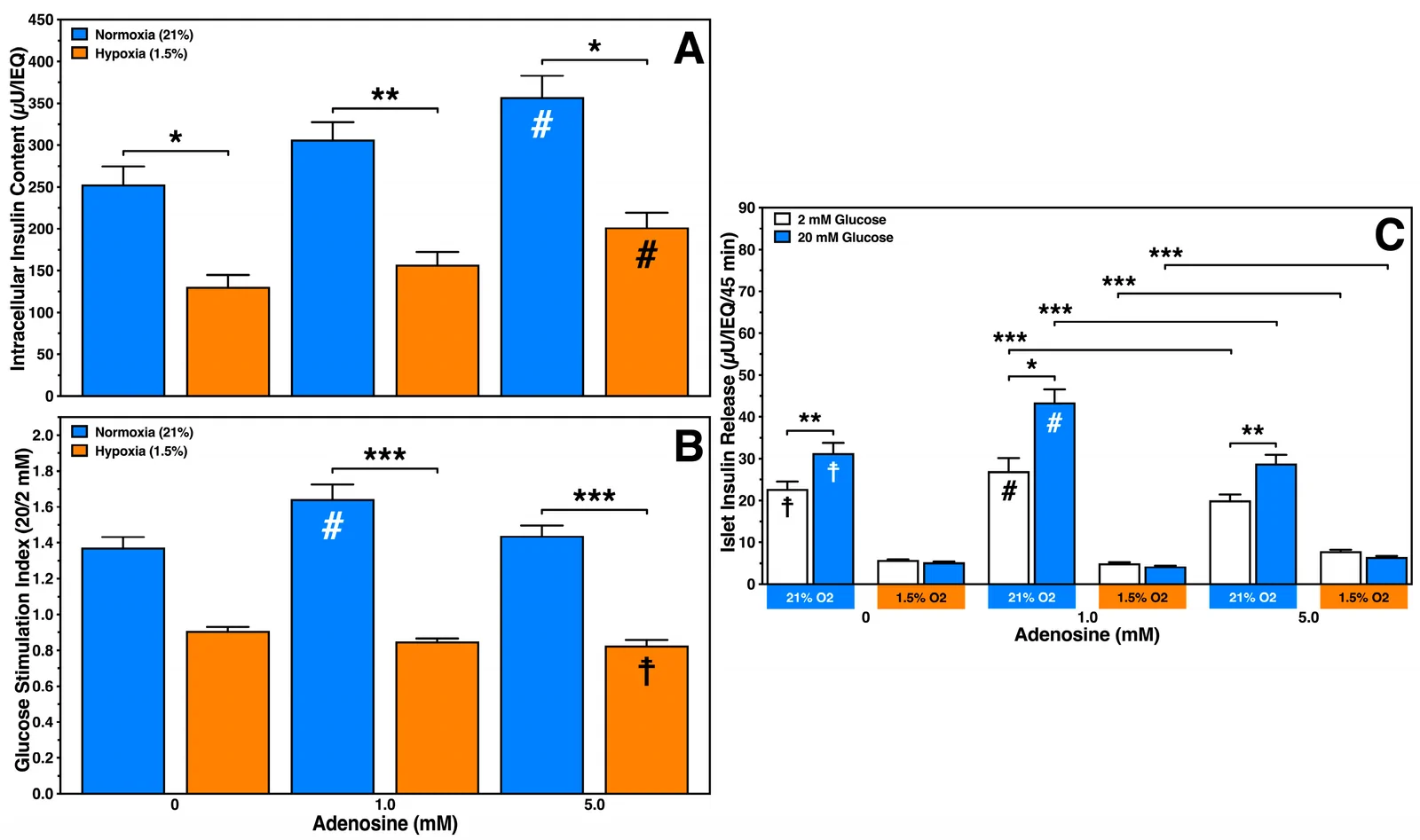
Effect of ADO treatment on the (**A**) intracellular insulin content (µU/IEQ), (**B**) secretory capacity determined as GSI and (**C**) insulin release (µU/IEQ) at 2 or 20 mmol/L glucose after 4–5 days of normoxic (21% O_2_) or hypoxic (1.5% O_2_) culture at 37 °C (*n* = 6). Symbols inside bars indicate (**A**,**B**) ☨ *p* < 0.01 and # *p* < 0.001 for comparison of normoxic and hypoxic vs. corresponding islets treated with 0 mmol/L ADO or (**C**) for comparison vs. corresponding hypoxic islets. Lines/arrows indicate * *p* < 0.05, ** *p* < 0.01 and *** *p* < 0.001 for comparison of experimental groups.

The storage and release of human islet insulin is detailed in Figure 4. A substantial reduction in intracellular insulin by approximately 50% was noted when islets were exposed to an atmosphere of 1.5% O_2_ (Figure 4A). The loss of stored insulin could be reduced by treatment with 5 mmol/L ADO (*** *p* < 0.001 vs. 0 mmol/L ADO).

Under normoxic conditions, a physiological insulin response towards glucose stimulation was observed as reflected by a GSI larger than 1.37. As expected, hypoxia had a substantial inhibitory effect on the secretory function as demonstrated by a GSI less than 1.0 in hypoxic islets (Figure 4B). In contrast to normoxic islets, which responded with an increased GSI when treated with 1 mmol/L ADO (# *p* < 0.001 vs. 0 mmol/L ADO), hypoxic islets demonstrated a reduction in the secretory capacity even when treated with 5 mmol/L ADO (☨ *p* < 0.01 vs. 0 mmol/L ADO).

Comparing the secretory behaviour of normoxic and hypoxic islets, it became obvious that a physiological positive insulin response towards glucose stimulation is virtually absent under hypoxic conditions (Figure 4C). In hypoxic islets, the amount of secreted insulin decreased by more than 30% compared with normoxic islets when exposed to the basal glucose concentration of 2 mmol/L (Figure 4C), whilst the reduction accumulated to approximately 55% in the stimulatory phase of 20 mmol/L glucose. Under hypoxic conditions, the presence of 5 mmol/L caused a significantly higher basal and stimulated insulin release (Figure 4C, *** *p* < 0.001 vs. 1 mmol/L ADO). In contrast, when islets were cultured in 21% oxygen, the switch of glucose concentration from 2 to 20 mmol/L resulted in a significantly increased discharge of insulin (* *p* < 0.05, ** *p* < 0.01). In the normoxic atmosphere, the presence of 1 mmol/L ADO further increased the amount of basal and stimulated insulin secretion (Figure 4C, *** *p* < 0.001 vs. 5 mmol/L ADO).

## 4. Discussion

ADO is essential for the physiology and metabolism of all organs and tissues [1,2,3,4]. The function of ADO as a key stress signal in normal human physiology is particularly relevant during episodes of ischaemia or hypoxia [8,9,10]. During clinical pancreatic islet isolation, human islets experience considerable hypoxia, as well as enormous biophysical and biochemical stress, which substantially affects the functional and morphological integrity of the isolated islets [15,16,17,18,19]. The key determinants for the success or failure of islet transplantation are the yield, viability and functional potency of isolated islets. Therefore, one aim of the present study was to investigate the impact of ADO in relation to the key quality parameters of human islets, particularly under the consideration of the mRNA expression of different ADORA receptors.

### 4.1. ADORA mRNA Gene Expression

The expression of purinergic G-protein-coupled receptors had already been demonstrated in pancreatic tissue [25]. Amongst those, all four ADORA receptors (A1, A2a, A2b, A3) had been detected in human islets [32]. Our current experiments in human islets confirmed and further characterised these findings. The pattern of the mRNA expression of different ADORA receptors was very similar and varied only in a small range. Nevertheless, a subsequent study of Amisten et al. presented conflicting results by confirming the expression of ADORA3 in murine islets but not in human islets [44]. Interestingly, another study revealed that the affinity of ADORA3 is much higher in human than in rodent cell populations [24], which may have implications for the viability of human islets treated with 5 mmol/L ADO.

As demonstrated in different organs and tissues, ischaemia and hypoxia are the most relevant stress-related variables that affect the level of ADO and the expression of different ADORA receptors [4,8,9]. In agreement with these previous studies, we found that the mRNA expression of ADORA1, A2a, A2b and A3 receptors was nearly doubled when human islets had been exposed to a hypoxic atmosphere. The level of ADORA mRNA expression was even further increased when hypoxic islets were additionally treated with ADO. No significant effect of ADO on ADORA receptor expression was observed in normoxic human islets. These findings indicate that ADO can indeed amplify the ADORA receptor expression in a hypoxic environment but is incapable of increasing ADORA receptor expression in human islets per se.

### 4.2. Islet Morphological Integrity and Mortality

Importantly, our study demonstrated that the lethal impact of hypoxia on human islet viability and morphological integrity is significantly ameliorated by adding a concentration of 1 mmol/L ADO, which also improved the viability and integrity of normoxic human islets in comparison with ADO-untreated islets. Our observations are in close compliance with a very recent study about human islets treated for 24 h with 1 mmol/L ADO. The approach of Perrier et al. followed a preconditioning strategy by exposing human islets to harmful insults after the withdrawal of ADO [45]. Their findings clearly suggested that a temporary treatment with ADO has a long-lasting protective effect on human islet viability and in vitro function even after the withdrawal of ADO, which may have implications for subsequent islet function after transplantation. The authors attributed this protection to the suppression of islet cell metabolism mediated by the activation of the ADORA1 receptor. Nevertheless, our PCR data clearly revealed that 5 mmol/L ADO induced an ADORA1 mRNA expression that is higher compared with 1 mmol/L ADO. The same observation was also made by measuring ADORA2a, −2b and −3, showing the highest mRNA expression after using 5 mmol/L ADO. Since an ADO concentration of 5 mmol/L diminished the protective effects on human islet viability, and morphological and functional integrity, it appears to be very probable that ADO causes a broad spectrum of effects ranging from islet protection to islet damage which affect the overall survival rate of human islets. Previous experiments in mice indicated that stimulation of the ADORA3 receptor causes a substantial reduction in initial viability [28]. In this context, it has to be discussed whether the decrease in viability in human islets can be related to the specifically high affinity of the human ADORA3 receptor towards ADO [24].

Our findings suggest that the detrimental effects of ADO are also relevant for the induction of apoptosis. This proapoptotic impact seemed to be correlated with increased ADO concentrations. Previous studies underlined the ability of hypoxia to induce islet destruction via both necrotic and apoptotic pathways [46,47,48]. Our investigations showed that the mortality of ADO-treated islets can be equally attributed to early apoptosis and to necrosis, appearing to be of similar relevance. This observation is in contradiction to previous experiments in EndoC–betaH1 beta-cells showing that high ADO concentrations stimulated apoptosis to a larger extent in comparison with necrotic cell death [49].

### 4.3. Islet Secretory Capacity

As demonstrated in a significant study with perifused rat and canine islets, a reduction in oxygen supply below a certain threshold substantially reduces the insulin release after glucose stimulation [50,51]. In a high degree of conformity with the observations of Dionne et al., we measured a significant drop in glucose-stimulated insulin secretion by approximately 90% when human islets were exposed to a pO_2_ of 10 mm Hg, with a concomitant loss of the insulin response to changes in the glucose concentration after switching from low to high glucose concentrations. When the hypoxic human islets were treated with 5 mmol/L ADO, a small, but nevertheless significant, increment in the basal and stimulated insulin release could be measured. However, the normal physiological insulin response towards glucose challenge could not be re-established in human islets treated with ADO.

Previous experiments in rats clearly demonstrated that ADO mediates a concentration-dependent effect on the kinetics of insulin release. Whilst ADO concentrations of ≤ 100 µmol/L decreased stimulated insulin release, concentrations of 1000 µmol/L or higher caused a stimulatory effect [30,52,53]. These observations match with findings in isolated mouse islets [28,54,55,56], but the higher sensitivity of murine species for the ADORA receptors generally requires lower concentrations of ADO [53]. The responsiveness of different ADORA receptors varied between the different subtypes and depends on the extracellular ADO level. Metabolic stress such as ischaemia or hypoxia causes an accumulation of ADO [57,58,59] that benefits low-affinity receptors such as ADORA2a and ADORA2b, promoting the formation of cyclic AMP (cAMP) and subsequent secretion of glucose-stimulated insulin [26,31,32,52,60,61]. In contrast, basal metabolic conditions favour high-affinity receptors like ADORA1 and ADORA3 [60,61,62]. The currently available data clearly suggest that ADORA1 mediates an inhibitory effect on insulin secretion [26,27,28,29,30]. An opposite effect was found after the stimulation of ADORA2a that seems to potentiate stimulated insulin release [28,62,63].

During the normoxic experiments, we noticed that the insulin response of human islets to ADO differs markedly from that of rodent islets. Whereas a concentration of 1 mmol/L of ADO enhances the stimulated insulin secretion of human islets, thereby corresponding to the secretory behaviour of rodent islets as discussed above [30,52,53,54,55,56], 5 mmol/L of ADO significantly diminished this augmentation. So far, we can only speculate whether this atypical ADO-mediated secretory behaviour of human islets might be related to their particular cellular organisation and architecture [64,65].

### 4.4. Limitations of the Study

ADO has been considered a stress key signal for human physiology and metabolism [8,9,10]. When islets are experiencing a severe lack of oxygen, the imbalance between adenosine triphosphate (ATP) synthesis and the fast consumption of adenosine diphosphate (ADP) and monophosphate (AMP) in vital metabolic processes quickly results in the production of purine nucleosides including inosine, hypoxanthine and ADO in the intracellular space, which quickly equilibrates with the extracellular space [9,10,11,24,57,58]. The high level of ADO can alter the expression and activity of the four different ADORA receptors [58,59,62]. Nevertheless, there is still a need for a debate to clarify whether these observations have implications for human islets since neither murine nor human islets have the enzymatic ability to convert exogenous AMP into ADO, in contrast to rat islets [66,67].

In this study, human islets were treated with an increment of 1 or 5 mmol/L ADO, which corresponds with the original formulation of UW solution still representing the gold standard for organ preservation and subsequent cold storage [68]. The results of the present study may serve as an opportunity to initiate a comprehensive discussion about the potential toxicity of an ADO concentration of 5 mmol/L, which may globally affect the preservation and cold storage of human donor organs.

To answer the important question of whether these ADO concentrations have the capacity to stimulate all four ADORA receptors at the same time, even those with the lowest sensitivity such as ADORA2a and ADORA2b [58,59,62], further detailed experiments are required to lock or unlock the different ADORA receptors and to identify their specific effects on morphological and functional islet integrity as well as mortality. In this regard, it may be worth discussing the relevance of the mRNA expression of certain biological substances. Previous studies clearly demonstrated that the correlation between mRNA and protein expression is quite low, due to the different production rates of mRNA and proteins [69,70,71] and the enormously varying half-lives of proteins [72]. For that reason, a separate study should be initiated in the near future to focus on the protein expression of different ADORA receptors.

The present study clearly shows that the findings are conflicting with respect to the protective effect of ADO on islet yield and overall survival on the one hand and increased islet mortality on the other. This critical dispute is aggravated by the complexity of apoptotic pathways, which are characterised by a significant overlap between the numerous variations of regulated cell death [73]. In this context, the impact of secondary necrosis should be considered. At the current time point, we can only speculate whether our data reflect a delay in necrotic cell death which may potentially increase during the following course of treatment.

## 5. Conclusions

In conclusion, this study demonstrated that all four ADORA receptors (A1, A2a, A2b, A3) are expressed in isolated human islets. The gene expression of the ADORA receptors is increased by hypoxia and further amplified by ADO treatment. No effect of ADO on ADORA receptor expression could be observed in normoxic human islets. As expected, hypoxia exerted a significant inhibitory impact on glucose-stimulated insulin secretion which could not be ameliorated by adding ADO. In contrast, supplementation with 1 mmol/L ADO significantly enhanced islet yield and secretory capacity but simultaneously enhanced early apoptosis, necrosis and total islet mortality.

In summary, our data suggest that ADO expresses an ambivalent character, with protective effects on islet overall survival on the one hand and a proapoptotic impact on the other.

## Figures and Tables

**Table 1:** Effect of different ADORA receptors in islet tissue retrieved from various species.

ADORA Receptor	ADO (Concentration)	Agent (Concentration)	Chemical Character	Physiological Effect	Species	References
**A1**	---	---		Increases insulin secretion	A1 deficiency mouse model	[26]
**A1**	0.01 mmol/L	---		Increases insulin secretion	A1 deficiency mouse model	[27]
**A1**	0.1 mmol/L	CPA (100 µmol/L)	Agonist	Increases insulin secretion	Mouse	[28]
**A1**	0.1 mmol/L	DPCPX (1 µmol/L)	Agonist	Potentiates insulin secretion	Mouse	[28]
**A1**	---	---		Increases insulin secretion	A1 deficiency mouse model	[29]
**A1**	1 mmol/L	DPCPX (10 µmol/L)	Antagonist	Potentiates insulin secretion	Rat	[30]
**A1**	---	DPCPX (1–50 µmol/L)	Antagonist	Increases insulin secretion	Rat	[31]
**A1**	---	GPCRs		Increases insulin secretion	Human	[32]
**A2a**	---	ATL146e (0.1 µmol/L)	Agonist	Reduces graft inflammation	Mouse	[33]
**A2a**	0.1 mmol/L	NECA (100 µmol/L)	Agonist	Increases insulin secretion	Mouse	[28]
**A2a**	0.1 mmol/L	CGS21680 (0.01 µmol/L)	Agonist	Increases insulin secretion	Mouse	[28]
**A2a**	0.1 mmol/L	CGS21680 (10–100 µmol/L)	Agonist	Reduces insulin secretion	Mouse	[28]
**A2b**	---	BAY 60-6583 (2 mg/kg BW)	Agonist	Improves glucose-homeostasis	Type 2 D.M. mouse model	[34]
**A3**	0.1 mmol/L	CI-IB-MECA (100 µmol/L)	Agonist	Increases insulin secretion	Mouse	[28]
**A3**	0.1 mmol/L	CI-IB-MECA (30 µmol/L)	Agonist	Reduces islet viability	Mouse	[28]

## Data Availability

Data are included within the article.
